# Effects of Teleassistance on the Quality of Life of People With Rare Neuromuscular Diseases According to Their Degree of Disability

**DOI:** 10.3389/fpsyg.2021.637413

**Published:** 2021-03-02

**Authors:** Oscar Martínez, Imanol Amayra, Juan Francisco López-Paz, Esther Lázaro, Patricia Caballero, Irune García, Alicia Aurora Rodríguez, Maitane García, Paula María Luna, Paula Pérez-Núñez, Jaume Barrera, Nicole Passi, Sarah Berrocoso, Manuel Pérez, Mohammad Al-Rashaida

**Affiliations:** Department of Personality, Evaluation and Psychological Treatment, Faculty of Psychology and Education, University of Deusto, Bilbao, Spain

**Keywords:** rare neuromuscular diseases, teleassistance, videoconferencing, psychosocial program, quality of life, disability

## Abstract

Rare neuromuscular diseases (RNMDs) are a group of pathologies characterized by a progressive loss of muscular strength, atrophy, fatigue, and other muscle-related symptoms, which affect quality of life (QoL) levels. The low prevalence, high geographical dispersion and disability of these individuals involve difficulties in accessing health and social care services. Teleassistance is presented as a useful tool to perform psychosocial interventions in these situations. The main aim of this research is to assess the effects of a teleassistance psychosocial program on the QoL levels of people with RNMDs who have different levels of disability. A sample of 73 participants was divided into an experimental group (*n* = 40), which participated in the intervention, and a control wait list group (*n* = 33). QoL was evaluated through the SIP and the SF-36, and disability through the WHO-DAS II. The participants with a moderate to severe level of disability were those who most benefited from the intervention. The results also revealed that the psychosocial teleassistance program was suitable to improve physical and psychosocial aspects of people suffering from a rare neuromuscular disease with a moderate level of disability, but just psychosocial aspects in those with a severe level of disability.

## Introduction

Rare neuromuscular diseases (RNMDs) are a group of hereditary or acquired diseases in which the motor neuron in the spinal cord, its axon, the neuromuscular junction and the muscle fibers innervated by the motor neuron may be affected, causing a progressive loss of muscle strength and the degeneration of muscles and the nerves that control them. Despite their complexity, they tend to share a number of characteristics, such as their chronicity and progressiveness, which may also be accompanied by dependency or disability, highlighting their chronicity. These diseases usually cause people who suffer them to require specialized, long-term care (ASEM, 2008; Amayra et al., 2014; Dowling et al., 2018; Iolascon et al., 2019).

Amongst the main signs and symptoms of RNMDs, weakness or loss of muscle strength are considered the most common symptoms, together with hypotonia, twitches, myotonia or muscle stiffness, decreased or absent muscle stretching reflexes, etc. (Bertorini, 2002).

People with rare neuromuscular disorders may have difficulties in performing certain activities of daily life, ranging from basic activities, such as eating or personal hygiene, instrumental activities (e.g., meal preparation, home maintenance, and traveling) to advanced activities (e.g., social interaction with peers). The symptoms that occur in many of these diseases, such as loss of muscle strength or rapid fatigue, can significantly affect the quality of life (QoL) of this population.

There are varying degrees of disability according to the disease and the stage of the disease the person is suffering. Generally, it is believed that those affected by a highly incapacitating and rapidly evolving RNMDs, such as amyotrohpic lateral sclerosis (ALS), will not enjoy life due to the physical symptoms the disease entails. However, many patients affected by ALS describe their life positively, although they physically deteriorate rapidly (Hardiman et al., 2004). It is here where other factors, such as emotional factors, play an important role in QoL (Hardiman et al., 2004; Cohen and Biesecker, 2010).

The QoL concept has been studied extensively in the literature. The World Health Organization defines QoL as the individual’s perception of their position in life within the context of the culture and values system in which they live and in relation to his/her objectives, expectations, standards, and concerns (WHOQoL Group, 1998).

Specifically, studies carried out with people suffering from RNMDs are scarce but still exhibit the interest in this concept (Grootenhuis et al., 2007; Twork et al., 2010; Boosman et al., 2011). For example, Twork, Wiesmeth, Klewer, Pöhlau, and Kugler carried out a study with 1518 patients suffering from myasthenia gravis (MG), which indicated that, although this groups’ life expectancy has been prolonged over the years, their health-related quality of life (HRQoL) was low, especially on issues related to difficulties in moving and depression (Twork et al., 2010).

Peric et al. (2010) also analyzed the HRQoL of two groups: one affected by ALS and other by myotonic dystrophy type 1 (MD1). Their results showed that both types of disorders had a similar disability in terms of the domains assessed through the SF-36 health questionnaire, with the exception of the bodily pain domain. They also indicated that the emotional state and the severity of the disease were factors that significantly influenced the HRQoL of those affected by MD1. In the case of ALS, the factors influencing the HRQoL were the severity of the disease and the educational level (Peric et al., 2010). In this sense, Graham et al. (2011) conducted a review of the literature on muscular disorders and how these affect QoL and their results indicated that aspects such as disease severity, pain, fatigue, and mood significantly affect the QoL of people suffering with various muscular disorders.

Grootenhuis et al. (2007) assessed the HRQoL of children and adults suffering from muscular dystrophy (Duchenne Muscular Dystrophy, Becker Muscular Dystrophy, and Limb Girdle Muscular Distrophy) and their results indicated that suffering one of these diseases adversely affects the HRQoL. For its part, Jacques et al. (2019) evaluated HRQoL in a sample of patients diagnosed with DMD, BMD, LGMD or Facioscapulohumeral muscular dystrophy (FSH). Compared to the control group without MD, all dimensions of HRQoL showed lower levels in patients with some form of MD.

In summary, the literature indicates that there is a growing interest in assessing the impact on the HRQoL in this group, in which it is reduced (Piccininni et al., 2004; Antonini et al., 2006; Twork et al., 2010; Graham et al., 2011). Thus, it is necessary to allocate part of the current research effort toward the study of the HRQoL of people with RNMDs and to take the emphasis off the purely physical level, taking into account the multidimensional impact of these diseases (Graham et al., 2011).

In relation to disability, there are several authors that consider this concept as a variable to study in their research on RNMDs (Nätterlund et al., 2000; Boström et al., 2005; Zebracki and Drotar, 2008; Kohler et al., 2009; Leonardi et al., 2009, 2010; Raggi et al., 2010; Kierkegaard et al., 2011). Diseases such as MG can produce long-term or permanent disability that will have serious repercussions on QoL (Leonardi et al., 2010). Thus, the main objective of the study carried out by these authors was to analyze the relationship between HRQoL and disability in a sample of MG’s patients. Their findings showed both a physical and mental decline. The decline in HRQoL and the degree of disability became more pronounced as the severity of the disease increased, that is, in those with bulbar symptomatology.

In this regard, it is noteworthy that the treatments available to treat some of the symptoms of MG and its early diagnosis have significantly reduced the mortality rate and the high disability associated with the disease in the past. However, despite the progress in these treatments, some symptoms do not subside in a certain number of patients in whom the disability is still present (Angelini, 2011).

Other RNMDs, such as muscular dystrophies, do not have any symptomatic treatments like those available for MG. The degree of disability in these diseases is very high, resulting in mobility problems (warranting the need to use wheelchairs), and a severe weakness of both the lower and upper musculature. This increased disability is linked to an increased dependency on others and to a decline in their HRQoL (Nätterlund et al., 2000), which may lead to the increase of anxiety levels and depressive symptoms. In this way, a psychosocial intervention should be appropriate in order to improve daily life of individuals with these diseases.

The literature about the psychosocial interventions, both face-to-face and remote, available for people affected with RNMDs is limited. This literature includes psychoeducative interventions, breathing and relaxation techniques, guided imagery, and problem-solving therapy, among others. Thus, De Freitas et al. focused on diaphragmatic breathing for people with MG and indicated an improvement in the breathing muscular strength, and the breathing pattern and resistance (De Freitas et al., 2005). In the case of the visual imagery, Collins and Dunn (Collins and Dunn, 2005) carried out a study about the relationship between the improvement of the arms strength and the rashes and hands pain related to dermatomyositis, and the practice of visual imagery and transcendental meditation. The results showed a significant relationship between these factors, and a recovery. Finally, Horowitz (2008) pointed out that the use of psychological treatments, such as problem-solving therapy, is appropriate to reduce the depressive levels of people with ALS.

Given the particular casuistry of individuals suffering from low prevalence neuromuscular diseases, such as physical and mobility difficulties or the high geographical dispersity of these diseases, and the difficult access to healthcare services for those living in rural areas, teleassistance is presented as a possible way to overcome such impediments. In this sense, two studies were able to improve several areas of QoL in people affected by rare neuromuscular disorders (Padua et al., 2009; Martínez et al., 2014). In the first study, individuals with BMD, FSH, MG, and other neuromuscular diseases of low prevalence, participated in a videoconferencing psychosocial program. The authors found improvements in QoL and disability (Martínez et al., 2014). In the second study, an online program for the improvement of social and emotional skills of patients with MG was developed. The results indicated that the intervention was appropriate for improving some aspects related to QoL, such as mental health or role limitations due to emotional problems (Padua et al., 2009).

Finally, Nelson et al. (2013) emphasized that videoconferencing can bridge the gap between the important need of many people to receive psychotherapy and the inaccessibility of existing interventions. In fact, according to these authors, despite advances in the treatment of many mental illnesses, many people do not have access to such interventions. Videoconferencing systems allow the therapist and the patient to communicate with each other in real time, being able to observe both verbal and non-verbal communication, which is similar to the relationship that would take place in a traditional face-to-face intervention (Nelson et al., 2013).

Thus, the objective of the present study is to evaluate the impact of a psychosocial teleassistance program on the improvement of the QoL of people affected with RNMDs, taking into account their degree of disability.

## Materials and Methods

### Participants

Based on the characteristics of the sample under analysis in this study, a convenience sampling method was carried out. Thus, the recruitment of the sample was conducted through various associations of patients affected by a rare neuromuscular disease (e.g., Myasthenia Association of Spain, AMES; Spanish Neuromuscular Disease Federation ASEM; etc.), and the Cruces University Hospital (Barakaldo) and the Basurto Hospital (Bilbao).

The initial sample was composed of a total of 80 participants with a rare neuromuscular disease, resident in various parts of Spain. These participants were divided into two groups: an experimental group, which received the psychosocial online intervention; and a wait list control group, which did not participate until the experimental group finished.

The inclusion criteria for the experimental group were as follows: (a) to be over 18 years of age; (b) to be diagnosed with a rare neuromuscular disease by a specialist; (c) to have access to a computer, webcam, microphone, speakers and an Internet connection in order to take part in the videoconference sessions; (d) to sign a written informed consent form for the participation in the evaluation process; and (e) to sign a written informed consent form for the participation in the intervention program. The exclusion criteria for this group were the following: (a) to be underage; (b) the presence of a severe psychopathology, according to criteria of the Diagnostic and Statistical Manual of Mental Disorders DSM-IV-TR; and (c) the presence of hearing or visual impairments that hinder the correct performance of the intervention’s activities.

As for the control group, their inclusion criteria were: (a) to be over 18 years of age; (b) to be diagnosed with a low prevalence neuromuscular disease by a specialist and (c) to sign a written informed consent for the participation in the evaluation process. Finally, the exclusion criteria for this group were: (a) to be underage; and (b) the presence of a severe psychopathology, according to criteria of the Diagnostic and Statistical Manual of Mental Disorders, DSM-IV-TR.

After eight participants abandoned the control group, the final sample consisted of 73 participants with a rare neuromuscular disease (Table 1), 40 from the experimental group and 33 in the control group.

**TABLE 1:** Sample distribution according to the type of neuromuscular disease.

**Type of neuromuscular disease**	**Experimental group**		**Control group**	
	**(*n* = 40)**		**(*n* = 33)**	
Myasthenia gravis	26	(65%)	22	(66.7%)
Facioscapulohumeral muscular dystrophy	2	(5%)	5	(15.2%)
Becker muscular distrophy	3	(7.5%)	0	(0%)
Limb–Girdle muscular dystrophy	5	(12.5%)	2	(6.1%)
Emery–Dreifuss muscular dystrophy	1	(2.5%)	0	(0%)
Spinal muscular atrophy	2	(5%)	1	(3%)
Charcot Marie Tooth	1	(2.5%)	0	(0%)
Dermatomyositis	0	(0%)	1	(3%)
Hereditary spastic paraparesis	0	(0%)	2	(6.1%)

With respect to the sociodemographic variables, the experimental group included 16 men and 24 women, aged between 19 and 73 years (47.23 ± 11.85). The control group consisted of 16 men and 17 women, aged between 27 and 79 years (55.52 ± 13.57).

Finally, the sample was divided attending to the degree of disability, according to data provided by the disability questionnaire of the World Health Organization WHO-DAS II and using the degree of disability scale provided by the International Classification of Functioning, Disability and Health. Therefore, four subgroups were formed in both the experimental group and the control group: no disability; mild degree of disability; moderate degree of disability; and severe degree of disability. There were no participants in either group with a degree of total disability.

### Instruments

An *ad hoc* semistructured interview was created with the purpose of gathering information on the most relevant socio-demographic data.

Subsequently, and with the aim of assessing the degree of disability of the sample, the World Health Organisation Disability Assessment Schedule II, *WHO-DAS II*, was used in its 36-item version. This instrument was developed by WHO, based on the CIF (Henao and Pérez, 2011). Its main objective is to assess disability, focusing on the difficulties (increased effort, discomfort or pain when performing certain activities, the need to perform such task more slowly or the need to change the way it is performed, for example, using a wheelchair for mobility) associated with various health conditions (diseases or health problems, injuries, mental or emotional problems, or problems associated with alcohol or drugs), taking into account the past 30 days.

It includes six domains: understanding and communicating, getting around, self-care, getting along with people, life activities, and participation in society. In turn, it provides an overall score taking into account the work/no work factor, and enabling the assessment of the degree of total disability in both people who work or study and those who do not.

As for the psychometric data of this instrument in its Spanish version, the data indicate that the WHO-DAS II presents an adequate internal consistency and test–retest reliability. Regarding the internal consistency, high Cronbach’s alpha values, greater than 0.70, are noted for different domains, and even higher for the global scales, that is, in the total number of people working and in the total not working (α = 0.93). Focusing on the test–retest reliability, the correlation coefficients of this instrument are greater than 0.80 in five domains and in both global scales, being 0.76 in the domain of getting along with people. The internal consistency in a subset of people with physical illnesses was also evaluated, reporting values higher than 0.73 in four of the domains, except for understanding and communicating (α = 0.64) and getting along with people (α = 0.57), and values of 0.93 for the global scales. The test–retest reliability continued to show high correlation values in four of the domains and in the global scales; however, once again, the understanding and communicating domain and the getting along with people domain presented moderate indexes of 0.65 and 0.72, respectively (Vázquez-Barquero et al., 2006).

In addition, in order to evaluate the QoL, the *Sickness Impact Profile* (SIP) and the *Health Questionnaire SF-36* instruments were used. The SIP developed by Bergner et al. (1976) and adapted by Badía and Alonso (1994), consists of 136 items which analyze the current health situation, and is divided into twelve different categories: ambulation, mobility, body care and movement, sleep and rest, eating, employment, home management, recreation and pastimes, social interaction, alertness behavior, emotional behavior, and communication. Regarding the psychometric data of the Spanish version, a high internal consistency (α = 0.95) is reported, being slightly lower in the categories of eating and sleep and rest. Moreover, it also shows a high test–retest reliability (*r* = 0.96) and an intraclass correlation coefficient of 0.95 (Badía and Alonso, 1994).

Furthermore, the SF-36 is an instrument designed to assess the QoL related to health in both the general population and in more specific groups, as well as to compare the weight of different diseases, to identify the benefits of a given intervention and to examine individual patients (Ware, 2000; Vilagut et al., 2005). The main objective of this test is to assess the health status of the person, both positively and negatively through 36 questions on eight dimensions related to health (Vilagut et al., 2005), which are: physical functioning, role limitations due to physical problems (role-physical), bodily pain, general health, vitality, social functioning, role limitations due to emotional problems (role-emotional), and mental health. With regard to the psychometric properties of the Spanish version of the SF-36, Alonso et al. (1995) reported a high internal consistency, with a Cronbach’s alpha coefficient greater than 0.70 for all dimensions except social functioning, where the coefficient obtained was 0.45. These authors conducted two administrations of the instrument, with a separation between them of 2 weeks, obtaining intraclass correlation coefficients ranging between 0.58 and 0.99 (Alonso et al., 1995). Additionally, Ayuso-Mateos et al. (1999) reported good internal consistency, with a Cronbach’s alpha of between 0.70 and 0.90 in all dimensions of the SF-36.

### Procedure

To carry out this study, a quasi-experimental pretest-posttest design with a non-equivalent control group was used.

The first phase consisted on the creation of a semi-structured interview containing questions related to socio-demographic and medical data. In addition, a psychosocial teleassistance program was developed with its respective sessions. This program was implemented through the Skype videoconferencing software. The psychosocial teleassistance program consisted of five blocks: psychoeducation, relaxation, emotional reactions, irrational beliefs and problem solving, which were addressed over seven sessions (Table 2), conducted by a psychologist.

**TABLE 2:** Sessions content.

**Session 1**	**Session 2**	**Session 3**	**Session 4**	**Session 5**	**Session 6**	**Session 7**
– Introduction. – General explanation of contents and objectives. – Assessment of expectations. – HomeWork: read the text: emotional reactions to the disease.	– Psychoeducation. – Working with the emotional reactions to the disease. – Discriminating between healthy and non-adaptive emotions. – HW: emotional worksheet.	– Reviewing and working with the worksheets. – Promoting the use of adaptive emotions. – Relaxation audio (3 times a week). – Breathing control. – HW: read the text: irrational beliefs.	– Reviewing the HW. – Working with irrational beliefs: – A-B-C model. – Identifying irrational beliefs. – HW: Thoughts and irrational beliefs worksheets.	– Working with the worksheets: identifying cognitive distortions. – Modifying irrational beliefs and replacing them with alternative ones. – HW: • TREC self-help questionnaire. • Read the text: problem solving strategies.	– Working with the questionnaire and resolving questions. – Explanation of the problem solving therapy and its five steps. – Using the steps in a problem pointed out by each participant. – HW: Apply the solution found.	– Using the steps in a problem pointed out by each participant (2nd part). – Encouraging participants to keep working with the exercises learned throughout the intervention. – Farewell and group closing.

In a second phase, the sample recruitment was carried out through contact with various associations of patients affected by rare diseases as well as through the Cruces university hospital and the Basurto Hospital (Bilbao).

The third phase included the initial interview, followed by the instruments used to assess disability and QoL. These evaluations were conducted in person and remotely. If participants had access to a computer and Internet, they were asked whether they wished to participate in the experimental group, and if they answered affirmatively, they were included in that group. If they did not have access to these resources or if they were not interested in taking part as experimental participants, they were included in the control group. All participants, both in the experimental group and in the control group, gave their informed consent to collaborate in the study.

In a fourth phase, before the start of the intervention process, Skype user profiles were created for each participant within the experimental group, subsequently informing these participants of such profiles. They were instructed on how to use Skype, in the case of not being familiar with the program, and how they should “log in” in order to start the group videoconference. Moreover, an email account was provided to each of these participants to receive weekly tasks.

The fifth phase consisted in carrying out the intervention procedure, which lasted 7 weeks, performing one session per week.

In a final phase, the post-assessment of the participants from both the experimental group and the control group was performed using the same instruments of the pre-test evaluation. The time between the pre- and post-test measures was approximately 2 months.

### Statistical Analyses

In order to perform a quantitative analysis of the data, the SPSS (*Statistical Package for the Social Sciences*) version 25 program was used. The adjustment to the normal distribution of the sample was analyzed for each of the variables studied, using the Kolmogorov–Smirnov test. Besides, homogeneity analyses of the sample were carried out between the experimental group and the control group through the use of the Chi-square test for categorical variables (most of the sociodemographic and medical variables assessed) and Mann–Whitney’s U non-parametric test for the variables of age, number of children, age at which symptoms began, age at which diagnosis was confirmed and the clinical variables of QoL and disability. Finally, in order to verify the change occurred between the pre-test measure and the post-test measure of the various subgroups established according to the different degrees of disability, the non-parametric test of Wilcoxon’s difference in medians was used, setting a value of *p* < 0.05 when statistically significant differences occurred between these measures. This was performed for both, the experimental and the control groups.

## Results

The results concerning the homogeneity of the sample indicated no statistically significant differences between the experimental and the control groups for most of the sociodemographical and medical variables analyzed, with the except of age variable (*U* = 435.50, *Z* = −2.490, *p* = 0.013). A detailed description of the characteristic of the study sample, both in the experimental group and in the control group, can be found in Table 3.

**TABLE 3:** Distribution of sociodemographical and medical variables.

**Variable**		**Experimental group (*n* = 40)**	**Control group (*n* = 33)**
Gender	Male	16 (40%)	16 (48.5%)
	Female	24 (60%)	17 (51.5%)
Marital status	Married	26 (65%)	20 (60.6%)
	In union	0 (0%)	3 (9.1%)
	Divorced	6 (15%)	2 (6.1%)
	Separated	1 (2.5%)	0 (0%)
	Single	6 (15%)	7 (21.2%)
	Widow/er	1 (2.5%)	1 (3%)
Children	Yes	24 (60%)	19 (57.6%)
	No	16 (40%)	14 (42.4%)
Level of education	Basic education level	7 (17.5%)	13 (39.4%)
	High school	12 (30%)	4 (12.1%)
	Vocational training	7 (17.5%)	2 (6.1%)
	Undergraduate degree	5 (12.5%)	5 (15.2%)
	Bachelor’s degree	9 (22.5%)	9 (27.3%)
Employment status	Employed	5 (12.5%)	7 (21.2%)
	Self-employed	1 (2.5%)	0 (0%)
	Unemployed (health)	6 (15%)	2 (6.1%)
	Unemployed (other reasons)	3 (7.5%)	4 (12.1%)
	Retired	5 (12.5%)	11 (33.3%)
	Homemaker	1 (2.5%)	3 (9.1%)
	Student	3 (7.5%)	0 (0%)
	Disability pension	16 (40%)	6 (18.2%)
Other non-neuromuscular diseases	Yes	19 (47.5%)	15 (45.5%)
	No	21 (52.5%)	18 (54.5%)
Medication intake	Yes	35 (87.5%)	24 (72.7%)
	No	5 (12.5%)	9 (27.3%)
Psychiatric or psychological support	Yes	6 (15%)	4 (12.1%)
	No	34 (85%)	29 (87.9%)
Diagnosis time delay	Less than 3 months	1 (2.5%)	5 (15.2%)
	From 3 to 6 months	6 (15%)	1 (3%)
	From 6 to 12 months	5 (12.5%)	7 (21.2%)
	From 1 to 2 years	4 (10%)	3 (9.1%)
	From 2 to 4 years	4 (10%)	4 (12.1%)
	From 4 to 6 years	3 (7.5%)	3 (9.1%)
	From 6 to 10 years	5 (12.5%)	3 (9.1%)
	More than 10 years	12 (30%)	7 (21.2%)

Statistically significant differences were found between both groups in the degree of disability, χ^2^(3) = 9.842, *p* = 0.020, being higher in the experimental group (Table 4).

**TABLE 4:** Degree of disability in both the experimental and the control groups.

**Degree of disability**	**Experimental group**		**Control group**	
	**(*n* = 40)**		**(*n* = 33)**	
No disability	5	(12.5%)	8	(24.2%)
Mild	10	(25%)	16	(48.5%)
Moderate	18	(45%)	8	(24.2%)
Severe	7	(17.5%)	1	(3%)
Total	0	(0%)	0	(0%)

Regarding the clinical variables of the pre-test, statistically significant differences were found in the QoL variables of mobility (*U* = 463.50, *Z* = −2.778, *p* = 0.005); social interaction (*U* = 473.00, *Z* = −2.123, *p* = 0.034); home management (*U* = 484.00, *Z* = −1.982, *p* = 0.047); physical dimension (*U* = 463.00, *Z* = −2.195, *p* = 0.028); and total SIP (*U* = 426.50, *Z* = −2.590, *p* = 0.010), on one hand, and the role-physical (*U* = 421.50, *Z* = −2.786, *p* = 0.005); social functioning (*U* = 356.50, *Z* = −3.455, *p* = 0.001); mental health (*U* = 463.50, *Z* = −2.184, *p* = 0.029); and the overall physical scale of the SF-36 (*U* = 478.00, *Z* = −2.017, *p* = 0.044), on the other. In this sense, the data indicate that the experimental group showed lower levels of QoL than the control group.

The pre-post test analysis showed significant differences in the experimental group (Table 5), specifically in social interaction (Z = −2.278, *p* = 0.023), with a mean score of 19.62 ± 19.68 in the pre-test and 13.87 ± 17.26 in the post-test; emotional behavior (Z = −2.229, *p* = 0.026), with a mean score of 17.50 ± 18.64 in the pre-test and 11.38 ± 17.87 in the post-test; and psychosocial dimension (Z = −2.117, *p* = 0.034), with a mean score of 18.48 ± 17.18 in the pre-test and 13.38 ± 14.08 in the post-test. Differences were also found in role-physical (*Z* = −2.882, *p* = 0.004), with a mean score of 35.62 ± 43.07 in the pre-test and 50.62 ± 45.10 in the post-test; and in mental component (*Z* = −1.949, *p* = 0.050), with a mean score of 44.67 ± 14.42 in the pre-test and 47.64 ± 12.89 in the post-test. In the control group differences were just found in ambulation (Z = −2.169, *p* = 0.030), with a mean score of 21.21 ± 18.52 in the pre-test and 17.67 ± 17.27 in the post-test time.

**TABLE 5:** Comparison between the overall pre-test and post-test measures of the experimental group.

	**SIP variables**	**Pre-test**	**Post-test**				**SF-36 variables**	**Pre-test**	**Post-test**			
		**Mean ± *SD***	**Mean ± *SD***	***n***	***Z***	***P-*value**		**Mean ± *SD***	**Mean ± *SD***	***n***	***Z***	***P*-value**
Physical	Body care and movement	21.63 ± 18.97	19.89 ± 17.41	40	–1.456	0.145	Physical functioning	42.00 ± 31.94	40.75 ± 31.89	40	–0.389	0.697
	Mobility	8.50 ± 13.69	9.25 ± 12.27	40	–0.404	0.686	Role-physical	35.62 ± 43.07	50.62 ± 45.10	40	–2.882	0.004**
	Ambulation	30.83 ± 22.10	28.54 ± 20.40	40	–1.280	0.201	Bodily pain	55.85 ± 35.30	55.77 ± 32.11	40	–0.102	0.919
Psychosocial	Social interaction	19.62 ± 19.68	13.87 ± 17.26	40	–2.278	0.023*	Social functioning	61.25 ± 30.59	64.06 ± 30.64	40	–0.884	0.377
	Emotional behavior	17.50 ± 18.64	11.38 ± 17.87	40	–2.229	0.026*	Role-emotional	59.16 ± 48.03	72.50 ± 42.62	40	–1.660	0.097
	Alertness behavior	24.50 ± 26.50	17.25 ± 22.75	40	–1.848	0.065	Mental health	63.60 ± 19.82	65.30 ± 18.52	40	–0.288	0.774
	Communication	10.27 ± 15.38	10.00 ± 16.07	40	–0.230	0.818						
Others	Sleep and rest	24.64 ± 20.45	20.71 ± 16.47	40	–1.092	0.275	Vitality	44.50 ± 22.75	49.75 ± 20.03	40	–1.935	0.053
	Home management	41.50 ± 28.96	39.25 ± 29.47	40	–0.930	0.352	General health	39.42 ± 21.48	41.62 ± 18.91	40	–1.208	0.227
	Recreation and pastimes	29.37 ± 26.63	24.06 ± 23.23	40	–1.445	0.148						
	Employment	10.27 ± 10.78	10.00 ± 10.31	40	–0.193	0.847						
	Eating	5.00 ± 8.32	5.27 ± 7.54	40	–0.406	0.684						
Global	Physical dimension	21.16 ± 16.18	19.83 ± 14.68	40	–0.882	0.378	Physical component	34.04 ± 9.81	34.50 ± 10.92	40	–0.027	0.979
	Psychosocial dimension	18.48 ± 17.18	13.38 ± 14.08	40	–2.117	0.034*	Mental component	44.67 ± 14.42	47.64 ± 12.89	40	–1.949	0.050*
	Total SIP	20.58 ± 13.65	17.66 ± 11.88	40	–1.608	0.108						

SD, standard deviation; n, number of participants; *p < 0.05, **p < 0.01.

Given the degree of disability of the participants, the found results demonstrate the existence of differences between the pre- and post-intervention assessments in some of the studied groups.

Firstly, regarding the participants in the experimental group who had no degree of disability (*n* = 5), and those with a mild degree of disability (*n* = 10), the comparison between the medians scores of the pre-test compared to those obtained in the posttest of these subgroups showed that no statistically significant change (*p* > 0.05) occurred for any of the variables of QoL.

Participants in the experimental group with a moderate degree of disability (*n* = 18) obtained statistically significant differences between the pre-test and post-test measures for several variables associated with QoL: in the psychosocial variable of the SIP questionnaire for social interaction (*Z* = −2.057, *p* = 0.040), as well as in some variables measured with the SF-36 questionnaire, such as the physical variable of role-physical (*Z* = −2.584, *p* = 0.010); in the psychosocial variable of role-emotional (*Z* = −2.066, *p* = 0.039); and in the vitality variable (*Z* = −2.069, *p* = 0.039). With respect to the mean scores in these variables, they were 25.55 ± 19.84 in the pre-test time and 18.61 ± 18.21 in the post-test time for the social interaction variable. In the case of the variable of role-physical, the average score in the pretest was 18.05 ± 32.99 and 43.05 ± 42.70 in the post-test. In the role-emotional variable, the average was 51.85 ± 48.80 in the pre-test measurement, obtaining a post-test value of 75.92 ± 42.48. Finally, the vitality variable obtained an average score of 36.94 ± 20.66 for the pre-test measurement, while the post-test measure reached the value of 45.00 ± 17.23 (Table 6).

**TABLE 6:** Comparison between the pre-test and the post-test measures of the participants with a moderate degree of disability.

	**SIP variables**	**Pre-test**	**Post-test**				**SF-36 variables**	**Pre-test**	**Post-test**			
		**Mean ± *SD***	**Mean ± *SD***	***n***	***Z***	***P-*value**		**Mean ± *SD***	**Mean ± *SD***	***n***	***Z***	***P*-value**
Physical	Body care and movement	25.36 ± 15.37	24.87 ± 14.36	18	–0.191	0.848	Physical functioning	29.16 ± 19.72	28.33 ± 24.00	18	–0.176	0.860
	Mobility	10.55 ± 13.04	12.77 ± 11.78	18	–0.791	0.429	Role-physical	18.05 ± 32.99	43.05 ± 42.70	18	–2.584	0.010**
	Ambulation	40.27 ± 16.72	37.96 ± 15.18	18	–0.790	0.430	Bodily pain	46.72 ± 34.30	50.44 ± 32.54	18	–0.549	0.583
Psychosocial	Social interaction	25.55 ± 19.84	18.61 ± 18.21	18	–2.057	0.040*	Social functioning	54.16 ± 29.07	54.86 ± 30.66	18	–0.106	0.916
	Emotional behavior	19.13 ± 20.09	15.43 ± 21.26	18	–1.182	0.237	Role-emotional	51.85 ± 48.80	75.92 ± 42.48	18	–2.066	0.039*
	Alertness behavior	33.88 ± 28.31	29.44 ± 27.75	18	–0.349	0.727	Mental health	60.66 ± 18.00	62.66 ± 19.06	18	–0.156	0.876
	Communication	12.34 ± 19.01	13.58 ± 19.63	18	–0.170	0.865						
Others	Sleep and rest	30.15 ± 17.59	26.98 ± 16.89	18	–0.733	0.463	Vitality	36.94 ± 20.66	45.00 ± 17.23	18	–2.069	0.039*
	Home management	55.00 ± 18.86	56.66 ± 19.09	18	–0.316	0.752	General health	33.05 ± 19.98	38.05 ± 21.36	18	–1.481	0.139
	Recreation and pastimes	37.50 ± 26.42	33.33 ± 23.87	18	–1.038	0.299						
	Employment	14.81 ± 10.08	11.11 ± 12.63	18	–1.285	0.199						
	Eating	4.93 ± 9.50	4.93 ± 7.83	18	–0.333	0.739						
Global	Physical dimension	26.04 ± 12.68	25.67 ± 11.12	18	–0.157	0.875	Physical component	28.84 ± 7.43	30.34 ± 8.58	18	–0.327	0.744
	Psychosocial dimension	23.61 ± 17.31	19.32 ± 16.35	18	–1.424	0.154	Mental component	43.61 ± 15.02	47.76 ± 12.19	18	–1.546	0.122
	Total SIP	26.06 ± 10.17	23.89 ± 10.17	18	–0.877	0.380						

SD, standard deviation; n, number of participants; *p < 0.05, **p < 0.01.

Taking into account the participants in the experimental group with a severe degree of disability (*n* = 7), the results obtained showed the existence of statistically significant differences for the psychosocial variable of social functioning of the SF-36 (*Z* = −2.236, *p* = 0.025). Considering the average scores on this variable, the pre-test measurement reached a score of 39.28 ± 24.39, being 48.21 ± 22.16 for the post-test measurement (Table 7).

**TABLE 7:** Comparison between the pre-test and the post-test measures of the participants with a severe degree of disability.

	**SIP variables**	**Pre-test**	**Post-test**				**SF-36 variables**	**Pre-test**	**Post-test**			
		**Mean ± *SD***	**Mean ± *SD***	***n***	***Z***	***P-*value**		**Mean ± *SD***	**Mean ± *SD***	***n***	***Z***	***P*-value**
Physical	Body care and movement	45.34 ± 7.88	38.50 ± 11.88	7	–1.841	0.066	Physical functioning	12.14 ± 12.53	17.85 ± 18.89	7	–1.890	0.059
	Mobility	17.14 ± 21.38	8.57 ± 15.73	7	–1.134	0.257	Role-physical	10.71 ± 28.34	28.57 ± 39.33	7	–1.633	0.102
	Ambulation	42.85 ± 16.26	41.66 ± 9.62	7	0.000	1.000	Bodily pain	47.85 ± 43.42	37.28 ± 38.85	7	–0.136	0.892
Psychosocial	Social interaction	31.42 ± 23.40	16.42 ± 17.49	7	–1.103	0.270	Social functioning	39.28 ± 24.39	48.21 ± 22.16	7	–2.236	0.025*
	Emotional behavior	26.98 ± 20.14	6.34 ± 8.74	7	–1.913	0.056	Role-emotional	19.04 ± 37.79	57.14 ± 46.00	7	–1.841	0.066
	Alertness behavior	32.85 ± 30.39	17.14 ± 11.12	7	–1.219	0.223	Mental health	51.42 ± 15.73	64.57 ± 7.80	7	–1.787	0.074
	Communication	15.87 ± 12.59	9.52 ± 7.66	7	–0.743	0.458						
Others	Sleep and rest	26.53 ± 22.47	20.40 ± 16.19	7	–0.966	0.334	Vitality	34.28 ± 19.88	41.42 ± 20.55	7	–0.738	0.461
	Home management	67.14 ± 14.96	62.85 ± 11.12	7	–0.535	0.593	General health	34.85 ± 24.90	39.42 ± 12.38	7	–0.542	0.588
	Recreation and pastimes	35.71 ± 30.12	33.92 ± 15.66	7	–0.105	0.916						
	Employment	9.52 ± 4.19	11.11 ± 0.00	7	–1.000	0.317						
	Eating	7.93 ± 8.39	7.93 ± 8.39	7	0.000	1.000						
Global	Physical dimension	38.41 ± 6.11	32.69 ± 9.40	7	–1.156	0.248	Physical component	29.03 ± 6.39	26.80 ± 7.25	7	–1.014	0.310
	Psychosocial dimension	27.97 ± 20.33	13.39 ± 9.76	7	–1.363	0.173	Mental component	35.28 ± 11.50	46.11 ± 10.56	7	–1.859	0.063
	Total SIP	32.14 ± 11.92	24.47 ± 6.95	7	–0.847	0.397						

SD, standard deviation; n, number of participants; *p < 0.05.

Regarding the control group, participants who did not show any degree of disability (*n* = 8) did not yield any statistically significant differences (*p* > 0.05) between the pre and post test measures.

Participants in the control group with a mild degree of disability (*n* = 16) showed statistically significant differences between the pre-test and the post-test measurements for the variable of mobility of the SIP (*Z* = −2.070, *p* = 0.038). The average score in the pre-test time was 21.87 ± 18.47, with 18.22 ± 18.05 being the post-test score.

In relation to the sample of participants in the control group with a moderate degree of disability (*n* = 8), the extracted data after the differences of medians between pre and post test measures showed the existence of statistically significant differences in two of the physical variables concerning QoL: in the body care and movement of the SIP questionnaire (*Z* = −2.217, *p* = 0.027) and in the physical functioning variable of the SF-36 (*Z* = −2.041, *p* = 0.041). Regarding the mean scores, the body care and movement variable obtained a score of 29.34 ± 18.11 in the pre-test measurement and 20.10 ± 19.15 in the post-test. As for the physical functioning variable, the average score reached the value of 13.75 ± 12.17 in the pre-test and 25.62 ± 25.69 in the post-test.

Finally, when taking into account the participants in the control group with a severe degree of disability (*n* = 1), there were not enough cases to carry out the appropriate analysis in order to check the difference in medians between pre and post test data.

## Discussion

The aim of the present study was to analyze the effects of a psychosocial teleassistance program on the levels of QoL of a sample of people suffering from a rare neuromuscular disease and with varying degrees of disability.

The symptomatology associated with these type of diseases, such as loss of muscle strength, muscle degeneration or fatigue, can have a serious impact on QoL or on the disability of a person (Grootenhuis et al., 2007; Leonardi et al., 2009; Twork et al., 2010; Graham et al., 2011; Jacques et al., 2019), as well as favoring the appearance of psychological problems due to its chronic nature (Schwartz et al., 1991; Jacques et al., 2019).

All these health consequences should be addressed adequately to reduce their impact on the living conditions of the individual. To do this, a number of therapeutic tools are set in place, such as psychoeducation, relaxation techniques and cognitive restructuring, which impact on cognitive, behavioral and emotional areas of the person and improve various aspects of their life so that they can function in their daily lives independently from the disease. The literature indicates the suitability of this type of interventions in improving the QoL of many people with various chronic (Rehse and Pukrop, 2003) and neuromuscular (Martín, 2010; Boosman et al., 2011; Martínez et al., 2014) diseases.

The particular characteristics of the group of people suffering from RNMDs, such as high geographic dispersion, the low frequency of the diseases or their associated mobility problems, makes the use of new technologies (in the case of the present study: Internet, videoconferencing and e-mail) ideal to implement a psychosocial program to improve all the mentioned aspects.

The findings of this study show that the participation in the teleassistance program improved QoL levels of individuals with RNMDs. Specifically, the levels of QoL of the participants in the experimental group with a moderate and severe degree of disability were improved after participating in the teleassistance psychosocial program, finding no changes in those without disability or suffering from mild disability.

One of the most important findings regarding these data is related to the course of such improvements in the QoL. In fact, when the degree of disability is moderate, this increase occurs in both physical and psychosocial variables, but only occurs for the psychosocial level when the degree of disability becomes severe.

This difficulty in improving the physical aspects when the degree of disability of the patient worsens has been reported in the literature, where it is evident that the greater the clinical implication of the disease and the degree of disability, the worse the physical levels of QoL become (Padua et al., 2009; Stefane et al., 2013). Moreover, it has been noted that the psychological and emotional functioning appear to be relatively independent of the severity of the disease (Graham et al., 2011), which could explain the presence of changes to purely psychological level in the subgroup with a severe degree of disability. In this regard, Jacques et al. (2019) stablishes that symptoms such as muscular weakness do not define QoL in people with muscular dystrophies.

Specifically, in the case of the subsample of participants in the experimental group with a moderate degree of disability, the improvement in the levels of QoL occurred in the physical variable of role-physical, in the psychosocial variables of social interaction and role-emotional, and in the vitality variable. Thus, in the first place, after the intervention, this subgroup of participants was less limited by physical problems related to their health when carrying out their daily activities. Secondly, the impact of the person’s health on their ability to relate to others was lower after participating in the teleassistance program, having, in turn, fewer limitations due to emotional problems. Finally, participants in this subgroup reported feeling energetic and dynamic more frequently versus tired or exhausted after the intervention program had ended.

Furthermore, with regard to the participants in the experimental subgroup with a severe degree of disability, the improvement of the QoL was focused on the psychosocial area, particularly in the social function variable. Therefore, the health status interfered to a lesser extent on the ability of the person to perform social activities after completing the teleassistance program.

The existing studies in the literature that use online psychosocial interventions to improve the levels of QoL in people with RNMDs are virtually non-existent. However, the research carried out by Martín (2010) with patients with MG is noteworthy, as it shares some of its results with those found in the present study. The study also notes an increase in the levels of QoL of the participants in their sample following the implementation of a program that addressed psychosocial aspects online.

The results obtained are consistent with those used in other studies that use remote interventions with individuals affected by more prevalent neuromuscular diseases, such as multiple sclerosis (Finlayson et al., 2011; Fischer et al., 2015).

Firstly, Finlayson et al. (2011)’s study focuses on videoconferencing as a tool to implement a group program to manage fatigue, with various psychoeducational elements, in people suffering from multiple sclerosis. The findings of these authors reflect an improvement in the QoL of participants receiving the intervention program, specifically in the role-physical variable of the SF-36 questionnaire, in comparison to the control group (Finlayson et al., 2011).

Secondly, in the study of Fischer et al. (2015), an improvement in the levels of QoL of its participants was observed upon application of an online intervention. This intervention used techniques such as psychoeducation, behavioral modifications, relaxation and problem solving, among others (Fischer et al., 2015). These psychosocial techniques were also used in the teleassistance program developed in this study.

Similarly, this improvement in the levels of QoL is reflected in several studies with various chronic diseases, which use distance learning systems to perform their respective interventions (Shepherd et al., 2006; Macea et al., 2010; Buhrman et al., 2011). Although these studies do not focus on the study of RNMDs, they take into account other diseases that share their chronic nature and, therefore, some of the consequences at the physical and psychosocial level, such as fatigue (De Vries et al., 2010).

Considering the degree of disability of the participants in this study, the mentioned results are consistent with those reported by Cosio et al. (2011), which report the presence of increased levels of QoL of a sample of people with multiple sclerosis, depression and disability after participating in a cognitive behavioral intervention program performed remotely (via telephone). It should be noted that this program shares several similarities with the one conducted in the present study, as it includes, among others, cognitive restructuring and problem solving techniques.

Similarly, they conform to those expressed by Van Groenestijn et al. (2015), which indicate an improvement in the QoL after a cognitive-behavioral intervention on patients affected by ALS, which were also psychologically distressed.

The improvement in the vitality variable which arises among the experimental participants with a moderate degree of disability coincides with the result found in the study of Visschedijk et al. (2004), which carried out a cognitive behavioral group intervention, even though it did not focus on the use of new technologies. In this sense, if the degree of disability of the participants included in their sample is observed, it is evident that this level was also moderate.

Furthermore, in the control group, an improvement in the physical levels of QoL was seen, particularly in participants with a mild to moderate degree of disability. In the first case, the change occurred in the mobility variable, despite not being very affected in the pre-test measurement. In the second case, that of the subgroup with moderate disabilities, improvements occurred in the areas of body care and movement, and in physical functioning. In the overall sample changes happened in ambulation.

In the case of the subsample with a mild degree of disability, in which most of the controls participants were included, the differences may have been due to the lower level of disability of the participants and therefore, the lower severity of the disease, which can affect change at the physical level of the individual (Graham et al., 2011). Besides, the subsample of participants with a moderate degree of disability was composed of a small number of subjects (*n* = 8), of which 4 participants approached the mild degree of disability, which might be the cause of the observed change, since, as noted, the severity of the condition suffered by the person negatively correlates to QoL (Antonini et al., 2006).

Certain limitations that occurred in this study should be considered. First, some of the characteristics of the group under study, such as the low prevalence or the high geographical dispersion, entailed serious difficulties in recruiting the sample. Those features led to the inclusion of a small sample size. However, similar sample sizes can be found in other studies involving patients affected by various neuromuscular diseases (Padua et al., 2001; Winter et al., 2010). In addition, the estimate of the average prevalence of neuromuscular diseases is situated approximately one person in every 3,500 inhabitants (Amayra et al., 2014). Second, there were some technical problems such as network disconnections or occasional electrical blackouts, which resulted in the brief interruption of the sessions. Finally, the selected instruments to measure both the levels of QoL and the degree of disability were generic, thus may not have been sensitive enough to measure specific aspects of the analyzed diseases (Padua et al., 2001).

## Conclusion

The improvement in the levels of QoL and disability reflected in the results of the present study highlight the importance and usefulness of including psychosocial interventions, making use of new technologies such as teleassistance, in social and healthcare services aimed toward the care of people suffering from neuromuscular diseases. In this regard, the degree of disability of the person suffering from a neuromuscular disease should be taken into account when allocating psychosocial interventions through teleassistance. As the results of the present study show, people with a moderate degree of disability are those which most benefit from psychosocial teleassistance programs, followed by those suffering from a severe degree of disability. Therefore, it is important that both social services and researchers studying this topic take into account this finding when implementing an intervention program with this collective or in future studies within this area.

## Data Availability Statement

The datasets generated for this study are not readily available because the datasets belong to the University of Deusto, Spain. Requests to access the datasets should be directed to OM, oscar.martinez@deusto.es.

## Ethics Statement

The studies involving human participants were reviewed and approved by University of Deusto Ethics Committee, Avenida de las Universidades, 24, Bilbao, Spain. The patients/participants provided their written informed consent to participate in this study.

## Author Contributions

All authors listed have made a substantial, direct and intellectual contribution to the work, and approved it for publication.

## Conflict of Interest

The authors declare that the research was conducted in the absence of any commercial or financial relationships that could be construed as a potential conflict of interest.

## Abbreviations

ALS: amyotrohpic lateral sclerosis
BMD: Becker muscular dystrophy
DMD: Duchenne muscular dystrophy
FSH: Facioscapulohumeral muscular dystrophy
HRQoL: health-related quality of life
LGMD: limb–girdle muscular dystrophy
MG: myasthenia gravis
MD1: myotonic dystrophy type 1
QoL: quality of life
RNMDs: rare neuromuscular diseases
SIP: sickness impact profile
WHO-DAS II: World Health Organization disability assessment Schedule II.
